# Antifibrotics in COVID-19 Lung Disease: Let Us Stay Focused

**DOI:** 10.3389/fmed.2020.00539

**Published:** 2020-09-09

**Authors:** Sachin Chaudhary, Bhupinder Natt, Christian Bime, Kenneth S. Knox, Marilyn K. Glassberg

**Affiliations:** ^1^Interstitial Lung Disease Program, University of Arizona Colleges of Medicine, Tucson, AZ, United States; ^2^Banner-University Medicine Division, Phoenix, AZ, United States

**Keywords:** antifibrotics, COVID - 19, ARDS, fibrosis, SARS - CoV-2, SARS, MERS (Middle East respiratory syndrome)

## Abstract

After decades of research, two therapies for chronic fibrotic lung disease are now approved by the FDA, with dozens more anti-fibrotic therapies in the pipeline. A great deal of enthusiasm has been generated for the use of these drugs, which are by no means curative but clearly have a favorable impact on lung function decline over time. Amidst a flurry of newly developed and repurposed drugs to treat the coronavirus disease 2019 (COVID-19) and its accompanying acute respiratory distress syndrome (ARDS), few have emerged as effective. Historically, survivors of severe viral pneumonia and related acute lung injury with ARDS often have near full recovery of lung function. While the pathological findings of the lungs of patients with COVID-19 can be diverse, current reports have shown significant lung fibrosis predominantly in autopsy studies. There is growing enthusiasm to study anti-fibrotic therapy for inevitable lung fibrosis, and clinical trials are underway using currently FDA-approved anti-fibrotic therapies. Given the relatively favorable outcomes of survivors of virus-mediated ARDS and the low prevalence of clinically meaningful lung fibrosis in survivors, this perspective examines if there is a rationale for testing these repurposed antifibrotic agents in COVID-19-associated lung disease.

## Introduction

The coronavirus 2 (SARS-CoV-2)-driven coronavirus disease 2019 (COVID-19) pandemic and its deadliest complication, acute respiratory distress syndrome (ARDS), have fundamentally changed our world. Clinicians are piecing together the puzzle that is COVID-19. Information on disease pathogenesis and possible therapies surfaced initially, much out of necessity, from social media, listservs, case reports, and non-peer reviewed observations. Now, months since the initial description in Wuhan, China, with almost 10 million infected worldwide and about half a million deaths, the clinical and the scientific communities have learned much and pivoted to high-quality evidence for the management of COVID-19 patients. Strong scientific rationale must be articulated before approaching critically ill, consent-weary patients and their families to enroll in clinical trials (1). Along the way, there have been a few missteps. A recent review has shed light on the potentially dangerous treatment decisions when equating ARDS seen in COVID-19 infection to the mechanistically distinct physiology of high-altitude pulmonary edema (2). Despite robust *in vitro* mechanistic rationale, hydroxychloroquine has failed to protect against respiratory viruses in previous studies (3) and yet again has not proven effective in COVID-19 (4, 5). Among the many excellent ongoing studies with good preclinical data in appropriate animal models, some arising directly from recent clinical observations, we were surprised to see studies proposing to use the FDA-approved anti-fibrotic therapies (nintedanib NCT04338802 and pirfenidone NCT04282902) for idiopathic pulmonary fibrosis (IPF) in COVID-19 patients. We acknowledge that some patients with severe, prolonged viral pneumonia and ARDS will die as a consequence of inflammation-induced fibrosis. We also recognize that clinical and experimental data suggest overlapping mechanistic pathways with inflammatory scar and IPF (6, 7). The survivors of ARDS, regardless of cause, clearly have important long-term limitations. Muscle weakness, exercise limitation, physical and psychological sequelae, and decreased physical quality of life are well known (8). However, lung function upon recovery is often normal or well preserved and improves over time (9), arguing against a need for fibrosis-preventive therapies. Therefore, COVID-19-associated lung fibrosis does not seem to be the next phase of this pandemic requiring preventive or curative interventions (10). In this review, we posit that, unlike patients with IPF, the COVID-19 survivors will follow a familiar course of intense pulmonary inflammation, leading to mild scarring and near-normal lung function recovery over time.

## Chronic Progressive Lung Fibrosis Is Not a Feature Among Survivors of ARDS

ARDS is a form of severe acute lung injury characterized by its acute onset, bilateral pulmonary infiltrates, severe hypoxemia, and noncardiac pulmonary edema. In most cases, this is accompanied by intense neutrophilic alveolitis (11). Mechanical ventilation is needed as supportive therapy for patients with ARDS and can perpetuate lung injury (12). ARDS is also characterized pathologically by an initial exudative and inflammatory phase, followed by a fibroproliferative phase and, in non-survivors, end-stage fibrotic lung. With supportive measures, including low-tidal-volume ventilation, to minimize ventilator-induced lung injury and fibrosis, ARDS outcomes are improved (13). Cabrera-Benitez describes a “fibrosis paradox,” where those patients who die of ARDS had a prolonged course and evidence of pulmonary fibrosis. In contrast, ARDS survivors have relatively little evidence of fibrosis when biomarker measurement, lung function testing, or imaging is performed (14).

Fibrosis on biopsy correlated with poor outcome in a diverse ARDS cohort, but most patients had mild to no fibrosis (15). In an autopsy study of ARDS, fibrosis was noted in 4% of patients with disease of <1 week in duration, 24% of patients with disease of 1–3 weeks in duration, and 14 of 23 patients with disease lasting longer than 3 weeks. Fibrosis was more frequent in ARDS of pulmonary origin than that of extrapulmonary origin (16). Nevertheless, survivors of ARDS have a favorable pulmonary prognosis. In one study, forced expiratory volume in the first second (FEV1), forced vital capacity (FVC), and lung diffusing capacity for carbon monoxide (DLCO) were mildly reduced, with >80% of survivors showing normal or mild abnormalities on chest imaging at 6-month follow-up (17). Herridge et al. followed patients for 5 years and noted normal or near-normal volumetric and spirometric test results by 5 years. The results of the 6-min walk tests were near normal. The most common finding in patients who had a chest CT available was minor, nondependent pulmonary fibrotic changes (9). Many of the studies enrolled patients at a time when low-tidal-volume ventilation, perhaps the best therapy available for preventing and treating ARDS, was in its early stages of being consistently employed in intensive care unit (ICU) care.

## Influenza-Induced ARDS

The majority of ARDS studies include a heterogeneous patient population in which the onset and the etiology of ARDS are ill defined. In contrast, the onset of a viral illness and its course are often known. Clinical, physiological, and radiological follow-up studies of patients with ARDS have been conducted in previous viral pandemics. Although it is difficult to tease out ARDS patients from ICU patients with severe viral pneumonias in the literature, studies of patients with ARDS due to influenza H1N1 and H7N9 strains have shown that, although functional impairment occurs, residual spirometric and radiological abnormalities are often inconsequential clinically, with evidence of distortion of septal lines, parenchymal bands, and bronchiectasis. Pulmonary function inevitably improves over time (18, 19). In some studies, mild diffusing abnormalities persist in ARDS patients despite the normalization of FEV1 and FVC (20).

## ARDS Induced by MERS And SARS

COVID-19, like other novel coronaviruses—severe acute respiratory syndrome (SARS, 2002 to 2004), Middle East respiratory syndrome (MERS, 2012–2015)—is associated with high mortality from ARDS and multi-organ system failure. Fewer studies are available for outcomes in MERS, but similar to other causes of viral-induced lung injury, MERS survivors have a reduced quality of life (21), and the pulmonary sequelae from MERS are mild. In a cohort of less severely ill MERS pneumonia patients, only the subgroup with severe pneumonia showed an abnormal mean diffusing capacity, which was mildly reduced at 68% of predicted normal (22). In a study of 36 patients with a median follow-up time of 43 days, the follow-up chest radiographs were normal in 64% of patients. Those with lung scarring (23) of varying degrees were older, and no patients were followed for 1 year to determine if those with acute findings had improved over time. In a few case reports of patients who died of MERS, the predominant finding at autopsy was diffuse alveolar damage (24).

Several longitudinal studies have examined the long-term outcomes in SARS survivors. It is estimated that up to 36% of patients with SARS required ICU admission, with 26% meeting the criteria for ARDS. These patients have a significant impairment in health status at 1 year, which show modest correlations but are out of proportion to near-normal pulmonary function. Less than 4% of patients had severely reduced DLCO, and none were hypoxemic on the 6-min walk test (25, 26). In a study by Xie et al., 20% of SARS survivors had residual radiographic abnormalities on follow-up. The findings included interstitial thickening, ground-glass opacification, bronchiectasis, and signs of volume loss. Forty patients underwent high-resolution computed tomography imaging examination after approximately 1 month, with over half of them showing an improvement (27). In another study, the predominant CT findings were air trapping and ground-glass opacities in 90% of patients. Reticulation and parenchymal bands were also common, followed by bronchiectasis in 20% of patients and honeycombing in one patient. In the subgroup of patients with ARDS, the ground-glass and interstitial opacity scores decreased significantly, although there was no significant change in air trapping at 4 to 5 months (28). In survivors of SARS followed for 15 years, pulmonary interstitial damage and functional decline caused by SARS mostly recovered within 2 years after rehabilitation (29). The histopathology of SARS has been extensively reviewed in autopsy series with limited information in SARS survivors. The lungs in SARS predominantly show diffuse alveolar damage and follow similar injury patterns, as seen in ARDS of other causes, with hyaline membranes and fibrinous tissue in alveolar spaces. The extent of fibrous organization correlates with the length of the disease. Active pulmonary injury, however, can be seen for months, and fibrin balls within airspaces with features of organizing pneumonia are unique (30).

## COVID-19 ARDS

Although it is too early to reliably define the long-term outcomes in patients recovering from a severe COVID-19 infection, patients with severe pneumonia have near-normal spirometry and moderate decreases in diffusing capacity (31). Radiographically, the viral lung injury shows patterns similar to SARS, with some patients developing predominant ground glass infiltrates evolving to linear bands and architectural distortion (32). COVID-19 is unique in that ARDS can be atypical, with severe hypoxemia at times being associated with near-normal respiratory system compliance in some patients. Despite sharing the same viral etiology, these severely hypoxemic patients may present quite differently, thus requiring different management algorithms (33). Even as these subtypes are being identified and histopathological studies are emerging, the exact mechanism of lung injury in COVID-19 remains unclear.

Autopsy data are now available from multiple centers. Common findings emerge from these reports, including DAD (the histopathological correlate of ARDS) at different stages in all patients (34). Thickened alveolar septa with perivascular lymphocytic–plasmocytic infiltration are common and reflect a viral etiology of ARDS. There are also novel findings showing enhanced microthrombi, endothelialitis, and vascular involvement in COVID-19 as compared to other etiologies of ARDS (35, 36). Until recently, one finding that has lacked emphasis, frequency, and consistency in these reports is pulmonary fibrosis. A recent systematic review by Polak et al. summarizes the pathological findings from both autopsy and biopsy reports. In lung samples from 131 patients, 17 were ante-mortem, including three lung transplant explants. The majority of these 17 patients did not survive to be discharged from the hospital. The histological patterns identified in the cohort were reactive epithelial changes with DAD in 85% and microvascular damage with microthrombi and organizing pneumonia in 59%. The fibrotic pattern was seen in 22% and occurred approximately 3 weeks after the illness, with 7% showing some evidence of microcystic honeycombing (37). Although it is clearly too early to comment on the long-term functional outcomes in COVID-19 patients, a personalized approach given the unique pathologic findings, physiology, and phenotypes is warranted (38). Nevertheless, we speculate that the lung function deficits will improve as recovery ensues and that survivors who develop lung scarring, much like other viral etiologies of ARDS, will overwhelmingly have minimal pulmonary physiological consequences.

## Putting Scarring and Lung Fibrosis Into Context

Our perspective is that the survivors of post-viral ARDS recover with mild residual pulmonary deficits and that interventions to prevent these mild abnormalities are unnecessary during the COVID-19 pandemic. Despite overlapping pathways, the timing, etiology, prognosis, and mechanistic underpinnings of post-viral scarring are quite different than chronic fibrosing interstitial lung disease. IPF is progressive and eventually fatal in most patients (39). Pulmonary fibrosis secondary to autoimmune causes such as rheumatoid arthritis and scleroderma may similarly progress and can be treated with an approved anti-fibrotic therapy. Patients with connective tissue diseases who develop lung fibrosis have a relatively poor prognosis (40). In contrast, while post-inflammatory changes can be seen in some ARDS survivors, progressive fibrosis has not been an important characteristic in ARDS related to respiratory infections and viral pneumonias.

Our recent understanding of the pauci-immune mechanism of IPF differs substantially from the intense inflammatory response noted in ARDS and viral pneumonias. Moreover, viral inflammation induces robust T cell responses that can persist for months (41). It is quite possible that a significant subset of patients with COVID-19 have ARDS physiology (or atypical ARDS with relatively normal lung compliance) due to high-intensity lymphocytic alveolitis. This contrasts with other causes of ARDS in which an intense neutrophilic alveolitis is the rule. Comparisons have been made between ARDS-related fibrosis in humans and the intense inflammation and scarring in the bleomycin mouse model, a model in which young mice resolve their fibrotic lung disease (42). Age and underlying lung diseases may be important risk factors for enhanced fibrotic responses following ARDS. In the late stages of ARDS, diffuse alveolar damage with excessive and abnormal deposition of extracellular collagen matrix predominates as a consequence of the known acute inflammatory insult. Interstitial and intra-alveolar fibrosis is often noted to varying degrees. The elevated levels of NT-PCP-III, which is derived from the cleavage of procollagen III, may be a useful biomarker to stratify therapies in critically ill patients with different phenotypes (43). Fibrosis from ARDS, in contrast to IPF, does not progress nor lead to a dominant pattern of honeycombing. Although the etiology of IPF remains obscure, the pathogenesis is best understood as a consequence of repetitive injuries followed by dysregulated repair processes, facilitated by telomere shortening, not intense inflammation (44–46).

## Discussion

An excellent and thought-provoking review by George et al. highlights many nuances related to the care of IPF patients in the context of COVID-19 (7). Caring for patients with an underlying fibrotic lung disease is complex. The currently available anti-fibrotics have pleiotropic effects, allowing for many hypotheses related to their potential utility in other disease processes. It is clear that studies will proliferate as commercial interests grow and the pandemic continues in the absence of effective anti-virals and vaccines. The currently approved anti-fibrotics are meant for chronic disease management and by no means are curative nor do they reverse fibrosis. As such, despite the enthusiasm to study these medications, we believe that there is insufficient scientific rationale to do so, given the favorable course and the low prevalence of clinically meaningful scarring in survivors.

The number of patients suffering from COVID-19 is accumulating and will be millions worldwide. Certainly we must evaluate patients, prospectively and retrospectively, to define the scope and the burden of residual pulmonary deficits and the fibrotic changes to determine their clinical significance. However, we find ourselves asking: Is it worth spending valuable time, resources, and scientific energy studying anti-fibrotic therapies in acutely ill, consent-weary patients that truly need a targeted antiviral treatment or trial? The responsible answer is “no.” Let us keep our focus during the pandemic.

## Data Availability Statement

The raw data supporting the conclusions of this article will be made available by the authors, without undue reservation.

## Author Contributions

All authors have contributed to the conception and design of the work, were involved in drafting and revising the content, gave final approval of the version to be published, and agree to be accountable for the integrity and all aspects of the work.

## Conflict of Interest

SC received consulting and speaking fees from Boehringer Ingelheim, Genentech, and Veracyte. MG serves on the advisory board for Bellerophon Therapeutics. The remaining authors declare that the research was conducted in the absence of any commercial or financial relationships that could be construed as a potential conflict of interest.
