# Corrigendum: Antifibrotics in COVID-19 Lung Disease: Let Us Stay Focused

**DOI:** 10.3389/fmed.2020.604640

**Published:** 2021-03-11

**Authors:** Sachin Chaudhary, Bhupinder Natt, Christian Bime, Kenneth S. Knox, Marilyn K. Glassberg

**Affiliations:** ^1^Interstitial Lung Disease Program, University of Arizona Colleges of Medicine, Tucson, AZ, United States; ^2^Banner-University Medicine Division, Phoenix, AZ, United States

**Keywords:** antifibrotics, COVID - 19, ARDS, fibrosis, SARS - CoV-2, SARS, MERS (Middle East respiratory syndrome)

In the published article, there was an error in affiliations for Kenneth S. Knox. Instead of having affiliations 1 and 2, Kenneth S. Knox should only have affiliation 2.

The authors apologize for this error and state that this does not change the scientific conclusions of the article in any way. The original article has been updated.

